# Phylogeographic insights into an irruptive pest outbreak

**DOI:** 10.1002/ece3.102

**Published:** 2012-05

**Authors:** Catherine I Cullingham, Amanda D Roe, Felix A H Sperling, David W Coltman

**Affiliations:** Department of Biological Sciences, CW405 Biological Sciences Building, University of AlbertaEdmonton, Alberta, T6G 2E9, Canada

**Keywords:** *Dendroctonus ponderosae*, forestry, irruptive populations, mountain pine beetle, phylogeography

## Abstract

Irruptive forest insect pests cause considerable ecological and economic damage, and their outbreaks have been increasing in frequency and severity. We use a phylogeographic approach to understand the location and progression of an outbreak by the MPB (*Dendroctonus ponderosae* Hopkins), an irruptive bark beetle that has caused unprecedented damage to lodgepole pine forests in western North America and is poised to expand its range across the boreal forest. We sampled MPB populations across British Columbia and Alberta and used phylogeographic methods to describe lineage diversification, characterize population structure, investigate expansion dynamics, and identify source populations of the outbreak. Using 1181 bp of mitochondrial DNA sequence from 267 individuals, we found high haplotype diversity, low nucleotide diversity, and limited lineage diversification. The overall pattern was consistent with isolation by distance at a continental scale, and with reduced diversity and population structure in the northerly, outbreak regions. Post-Pleistocene expansion was detected, however more recent expansion signals were not detected, potentially due to the size and rapid rate of range expansion. Based on the limited genetic structure, there were likely multiple source populations in southern British Columbia, although the magnitude of the demographic expansion and rate of spread have obscured the signature of these source populations. Our data highlight the need for caution in interpreting phylogeographic results for species with similar demographics.

## Introduction

**Figure 1:**
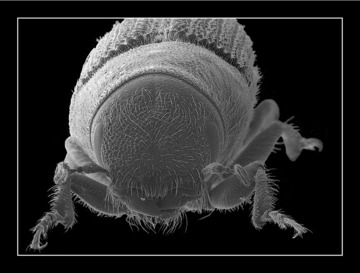
Scanned election microscopy image of mountain pine beetle (*Dendroctonus ponderosae*). Photo credit: Jack Scott.

The past few decades have seen an increase in the frequency and severity of insect pest outbreaks (Fleming and Candau 1998; Bale et al. 2002; Jepsen et al. 2008), often impacting host species of economic importance. Understanding the dynamics of an outbreak pest species is important from a management perspective. Knowing the source and spread of an outbreak can help delineate potential boundaries for control and provide the basis for developing strategies to prevent future outbreaks (Mun et al. 2003; Kim et al. 2006; Porretta et al. 2007). The use of population genetic methods to address management-associated questions for insect pest species is increasing. For example, Davies et al. (1999) determined that the source of a Californian outbreak of medfly (*Ceratitis capitata*) was Latin America. As well, Kobayashi et al. (2010) effectively used population genetic methods to determine that multiple source populations contributed to an outbreak of mirid bug (*Stenotus rubrovittatus*) in Japan, necessitating monitoring of nonoutbreak areas to prevent additional crop damage. Here, we use a phylogeographic approach to illuminate the outbreak dynamics of mountain pine beetle (MPB; Scolytidae: *Dendroctonus ponderosae* Hopkins, Fig. 1).

The MPB is a bark beetle that attacks many *Pinus* species in western North America including: *Pinus contorta* Dougl., *P. lambertiana* Dougl., *P. monticola* Dougl. Ed. E. Don, *P. ponderosae* P. Laws. Ex C. Laws, *P. albicaulis* Engelm., and *P. flexilis* James (Furniss and Schenk 1969; Safranyik and Carroll 2006; Safranyik et al. 2010). Recently, MPB populations have also been recorded in native stands of *P. banksiana* Lamb (Cullingham et al. 2011), an important boreal species in Canada and the eastern United States. While MPB are usually considered secondary pests that feed on suppressed or diseased trees, certain conditions can lead to irruptive population outbreaks. In these situations, MPB can successfully attack mature healthy trees, leading to significant stand mortality (Rudinsky 1962; Safranyik and Carroll 2006). These irruptive situations generally occur every 20–30 years and usually last for —five to seven years (Cole and Cahill 1976; Safranyik 2004; Taylor et al. 2006).

Outbreaks of MPB are important for the maintenance of healthy forest stands (Roe and Amman 1970; Logan and Powell 2001; Taylor and Carroll 2004), but anthropogenic factors have altered the dynamics of this system. Increasing global temperatures have prompted synchrony of beetle emergence in the summer and reduced winter mortality, thereby resulting in population increases (Hicke et al. 2006; Régnière and Bentz 2007; Powell and Bentz 2009). Population growth of MPB has also been fueled by an abundant food supply of continuous tracts of even-aged forest stands resulting from forest management practices (Taylor et al. 2006). All of these factors have contributed to the most severe MPB outbreak on record, and an unprecedented eastern expansion into northern Alberta outside of the historical range (Taylor and Carroll 2004; Raffa et al. 2008; Robertson et al. 2009; Bentz et al. 2010; Safranyik et al. 2010; Cullingham et al. 2011).

As MPB populations expand into novel habitats, we have been presented with an opportunity to explore their phylogeographic patterns in real time. Understanding how and where spatial expansion has occurred can assist with MPB management and help to predict risk of future spread. Phylogenetic analysis has been previously employed to analyze MPB populations in western Canada to understand historic demographics (Mock et al. 2007), although sampling was diffuse across the entire range, limiting the inferences that could be drawn about MPB phylogeography. Increased sampling within the outbreak populations would provide a more comprehensive understanding of the outbreak history. We are provided with a unique opportunity to apply expansion statistics to an outbreaking natural system. The range of MPB was affected by the Pleistocene glaciation due to range contraction and expansion of its host species caused by glaciation (Fazekas and Yeh 2006). In addition to this historic expansion, their range has grown considerably over the past 50 years into northern BC and west-central Alberta, representing a recent expansion. Here, we analyze MPB populations in British Columbia and Alberta, focusing on these areas of range expansion. We have included mitochondrial (mtDNA) data from Mock et al. (2007) to assess range-wide lineage diversification. The objectives of our study were to (1) assess lineage diversification and patterns of diversity in relation to geography across the MPB range, (2) to determine if signals of expansion can be detected in a natural system with complex population dynamics comprising historic and recent expansions, and (3) determine whether these data can be used to identify locations of source populations that may have contributed to the current outbreak.

## Methods

### Sample collection

**Figure 2:**
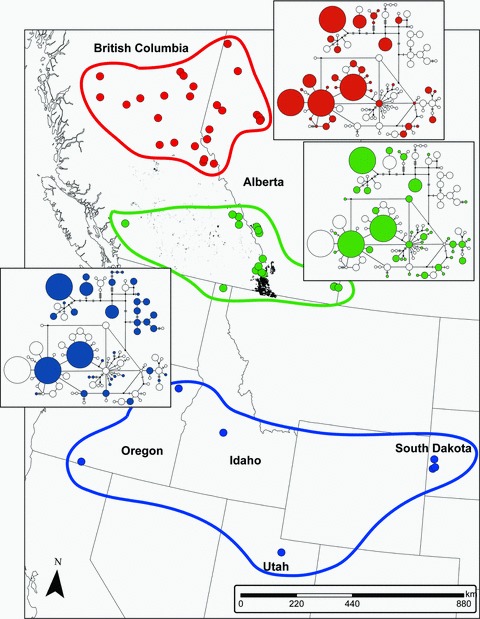
Sample sites, including data from Mock et al. (2007) divided into south (blue), central (green), and northern (red) groups. Beetle attack data for British Columbia and Alberta up to 1970 are included as shading as an indicator of the historic range postglaciation in Canada. The haplotype Median-Joining network for *D. ponderosae* is included, where the presence of a haplotype is indicated for each group. Circles are proportioned based on sample size. Sample sites in Arizona and California from Mock et al. (2007) are not shown. Beetle attack data are from Thandi and Taylor (unpubl. ms.).

Live beetles were collected from 26 locations in British Columbia, Alberta, and South Dakota (Table 1 and Fig. 2) from 2005 to 2009 prior to summer dispersal each year. Beetle larvae and adults were collected from different galleries by direct sampling from infested lodgepole pine and lodgepole pine × jack pine hybrid trees. For each tree, the Global Positioning System location was taken and collected beetles were transported to the laboratory on ice or in 95% ethanol and stored at –80°C upon arrival. We have also included in our analyses MPB samples analyzed by Mock et al. (2007) at eight localities: Arizona (F-AZ), British Columbia (FSJ-BC), California (R-CA, SB-CA), Idaho (BF-ID, S-ID), Oregon (L-OR), and Utah (K-UT) (GenBank: DQ865977.1–DQ866021.1; Table 1 and Fig. 2).

**Table 1:** Sample areas for *Dendroctonus ponderosae* mitochondrial DNA analysis with sample size (*N*), haplotype diversity (*H*), nucleotide diversity (π) (and associated standard deviation “SD”), their population assignment based population histories, and associated diversity measures. Sample areas in bold are taken from Mock et al. (2007).

Pop	Abbrev.	Year	*N*	*H*	SD	π	SD
North			173	0.9035	0.0109	0.0065	0.0034
Bowron Lake	BL	2006	13	0.8590	0.0886	0.0087	0.0048
Fox Creek	FC	2008	12	0.9242	0.0575	0.0057	0.0033
Francois Lake	FL	2006	11	0.8909	0.0740	0.0029	0.0018
Fairview	FV	2008	7	0.9048	0.1033	0.0082	0.0049
Grande Prairie	GP	2008	10	0.8444	0.0796	0.0044	0.0027
Houston	HO	2006	12	0.8636	0.0639	0.0014	0.0010
McBride	MB	2005	10	0.9778	0.0540	0.0083	0.0047
Mackenzie	MK	2005	9	0.8889	0.0910	0.0089	0.0051
Mount Robson	MR	2005	10	0.9556	0.0594	0.0085	0.0048
Prince George	PG	2005	9	0.9444	0.0702	0.0068	0.0040
Pine Pass	PP	2006	7	0.8095	0.1298	0.0047	0.0029
Quesnel	QN	2006	7	0.9048	0.1033	0.0101	0.0060
Telkwa	TK	2006	9	0.8889	0.0910	0.0058	0.0034
Tumbler Ridge	TR	2007	17	0.8309	0.0648	0.0035	0.0020
Valemount	V	2007	10	0.8444	0.1029	0.0089	0.0050
Willmore-Kakwa	WK	2008	11	0.9818	0.4630	0.0091	0.0051
**Ft St James**	**FSJ-BC**	**2003**	**9**	**0.2222**	**0.1662**	**0.0022**	**0.0015**
Central			78	0.9624	0.0106	0.0089	0.0045
Cypress Hills	CH	2007	12	0.9545	0.0569	0.0115	0.0063
Canmore	CA	07/08	16	0.9250	0.0389	0.0104	0.0056
Crowsnest Pass	CN	2008	12	0.9545	0.0569	0.0052	0.0030
Golden	G	2008	7	0.9524	0.0955	0.0109	0.0067
Kotonay-Yoho	KY	07/08	6	1.0000	0.0962	0.0094	0.0058
Sparwood	SW	2008	8	0.9643	0.0772	0.0082	0.0048
Whistler	WH	2006	8	1.0000	0.0625	0.0070	0.0042
**Bonner's Ferry**	**BF-ID**	**<2006**	**9**	**1.0000**	**0.0524**	**0.0694**	**0.0375**
South			87	0.9588	0.0096	0.0087	0.0045
Black Hills	BH	2009	11	0.8545	0.0852	0.0085	0.0047
Custer Peak	CU	2009	10	0.7556	0.1295	0.0054	0.0032
Tigervill	TV	2009	13	0.8462	0.0854	0.0054	0.0031
**Flagstaff**	**F-AZ**	**<2006**	**10**	**0.5333**	**0.0947**	**0.0108**	**0.0060**
**Kamas**	**K-UT**	**<2006**	**10**	**0.7778**	**0.1374**	**0.0849**	**0.0452**
**La Grande**	**L-OR**	**<2006**	**8**	**1.0000**	**0.0625**	**0.1082**	**0.0594**
**Klamath**	**R-CA**	**<2006**	**10**	**0.9556**	**0.0594**	**0.0834**	**0.0444**
**San Bernadino**	**SB-CA**	**<2006**	**8**	**0.7500**	**0.1391**	**0.0897**	**0.0492**
**Stanley**	**S-ID**	**<2006**	**7**	**0.7619**	**0.1148**	**0.0846**	**0.0476**

### DNA extraction and sequencing

Genomic DNA was isolated from whole beetle samples using one of two methods. The first method employed a standard phenol/chloroform procedure (Sambrook and Russell 2001) and following precipitation, DNA was suspended in Tris-EDTA (pH 8.0). Alternatively, a DNeasy Blood & Tissue Kit (Qiagen, Mississauga, Ontario) was used following manufacturer's instructions.

Partial mtDNA sequence was generated from portions of cytochrome *c* oxidase I (COI) and cytochrome *c* oxidase II (COII) genes as well as the complete tRNA-LEU using two sets of primers (C1-J-1718 (F): GGA GGA TTT GGA AAT TGA TTA GTT CC, C1-N-2611 (R): GCA AAA ACT GCA CCT ATT GA and C1-J-2193 (F): CCA GGG TTT GGT ATA ATT TCT T, C2-N-3174 (R): TTA GAG GGG AAG ACC TAT CTT GT; Simon et al. 1994; Cognato and Sperling 2000; Mock et al. 2007). PCR amplification was carried out in 25-ul reactions containing 25 ng DNA, 1× Thermopol buffer (NEB, Pickering, Ontario), 0.1 ng/µl bovine serum albumin fraction V (BSA), 4 mM MgCl_2_, 0.2 mM each dNTP, 0.25 µM each primer, and 1U Taq DNA polymerase (NEB). Thermocycling conditions consisted of an initial denaturation at 98°C for 30 sec, followed by 35 cycles of 95°C for 10 sec, 45°C for 30 sec, and 72°C for 60 sec, and a final extension at 72.0°C for 5 min. For primer pair C1-J-2193 and C2-N-3174, 15 ng DNA was used and the annealing temperature was 62°C. Primers and unincorporated dNTPs were removed using ExoSAP-IT (USB Corporation, Cleveland, OH) following manufacturer's instructions prior to sequencing. Purified PCR products were sequenced bidirectionally with Applied Biosystems Big Dye Terminator v 3.1 cycle sequencing kit (Applied Biosystems, Foster City, CA) using an Applied Biosystems 3730 DNA Analyzer. Sequence data were analyzed and assembled with Seqman (DNASTAR Lasergene, Madison, WI). Final assembled sequences were checked manually.

### Phylogenetic analyses

The most appropriate model of sequence evolution was selected using jModelTest 0.1.1 (Posada 2008) based on Akaike's information criterion. Using the parameter estimates, we calculated both a Maximum-Likelihood (ML) tree and a Median-Joining (MJ) network for the aligned mtDNA dataset. The ML tree was estimated using the on-line version of PhyML 3.0 (http://www.atgc-montpellier.fr/phyml/; Guindon and Gascuel 2003) using a general time reversible (GTR) model of nucleotide substitution, with the transition:transversion ratio, invariable sites, and gamma-shape parameter estimated from jModelTest, subtree pruning and regrafting for tree improvement with 10 starting trees, Shimodaira-Hasegawa like branch support, and 100 bootstrap replicates. We included *D. jeffreyi* Hopkins, sister species to *D. ponderosae* (Kelley and Farrell 1998) as an outgroup. The MJ network was calculated using Network 4.516 (fluxus-engineering.com). We implemented the maximum parsimony option to identify unnecessary median vectors and links (Polzin and Daneschmand 2003) and calculated the MJ network (Bandelt et al. 1999) using the output from this and the parameters from jModelTest.

Using a traditional phylogeographic approach (Avise 2004), we looked at lineage diversification in relation to geography across the range of the beetle. We divided our study area into three separate regions based on their biogeographic history (Fig. 2): (1) south—populations that were south of the Cordilleran ice sheet (Clark et al. 2009), (2) central—populations that are considered to have been endemic since post-Pleistocene expansions (Safranyik et al. 2010), (3) north—populations that have resulted from a recent range expansion of MPB (Fig. 2).

### Population structure

**Figure 3:**
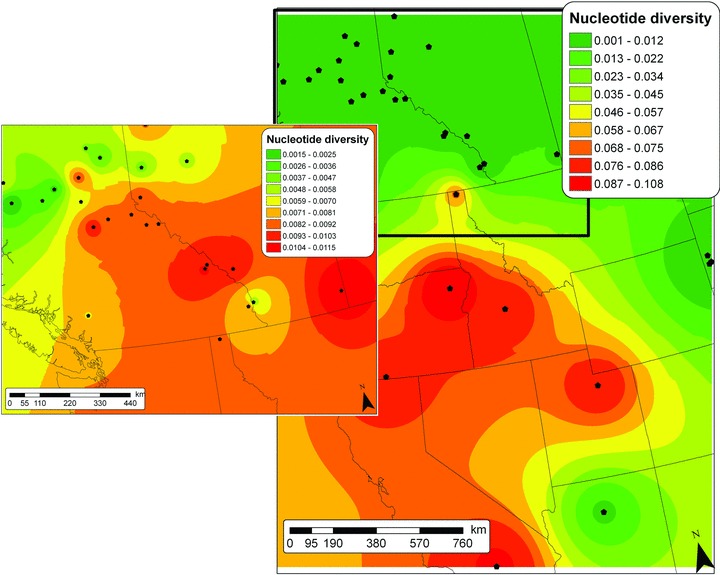
Spatially interpolated surface of mt nucleotide diversity for MPB populations across western North America, generated using inverse-distance weighting in the spatial analyst extension of ArcMap9.2. Inset shows the central and northern populations at a finer scale.

Given the evidence of post-Pleistocene colonization (Mock et al. 2007) and a more recent expansion, we calculated haplotype (*H*) and nucleotide (π) diversity for each subpopulation, and regressed these on latitude and longitude, where we expected diversity to decrease toward the north and to the east as MPB has recently expanded into central Alberta. All above calculations were performed in Arlequin 3.5.1.2 (Excoffier et al. 2005) and significance was estimated using 10,000 bootstrap replicates where appropriate. To test for signals of expansion, we estimated Fu's *F*s (Fu 1997), and R2 from Ramos-Onsins and Rozas (2002) given the statistical power of these statistics over other measures of expansion (Ramos-Onsins and Rozas 2002). Both measures were calculated in DnaSP v 5 (Librado and Rozas 2009) and significance was tested using the coalescent simulation to estimate the distribution. Using continuous surface maps, we visualized geographic patterns of diversity and signatures of expansion. The maps were generated in ArcMap 9.2 (ESRI) using the inverse-distance weighting (Fig. 3; Shepard 1968) calculation in the spatial analyst extension, using the six nearest neighbors for the estimation.

To examine the spatial genetic structure of MPB populations, we tested for isolation by distance including the Mock et al. (2007) data, and for just the outbreaking region in British Columbia and Alberta. We calculated pair-wise genetic differentiation using *F*_ST_ in Arlequin, which accounts only for haplotype frequency differences (Excoffier et al. 1992). Geographic distances were calculated among sample area centroids using Hawth's Tools (Beyer 2004) in ArcMap 9.2 (ESRI Inc, Redlands, CA). The correlation between the matrices was tested using a Mantel test implemented in zt (Bonnet and Van de Peer 2002) and significance was assessed using 10,000 iterations.

## Results

### Phylogenetic analyses

We obtained 1181 bp of mtDNA sequence from 267 individuals yielding 62 haplotypes (GenBank: JQ308436-JQ308479). We aligned our sequences with those from Mock et al. (2007) trimmed to our fragment size. This reduced the number of haplotypes described in Mock et al. (2007) to 35, which were analyzed with the 62 haplotypes from the newly sampled MPB populations. Most of the haplotypes were found in one of the three regions (south [31], central [23], and north [26]), 11 were shared between two of the regions (south–central [6], south–north [3], and central–north [2]), and six were shared among all regions. One hundred six variable sites were found, 97 transitions, seven transversions, and two transition/transversions. The ML tree was very shallow, characterized by short internal branches with little bootstrap support over 80% (Fig. S1). The low level of divergence among haplotypes was also reflected in the MJ network where there were some reticulations and a number of “star-burst” patterns (Fig. 2). Some phylogenetic resolution in both the MJ network and ML tree was found among haplotypes that occurred in the southern portion of the MBP range, although for the majority, lineage diversification did not correspond to geography (Fig. 2).

### Population structure

**Figure 4:**
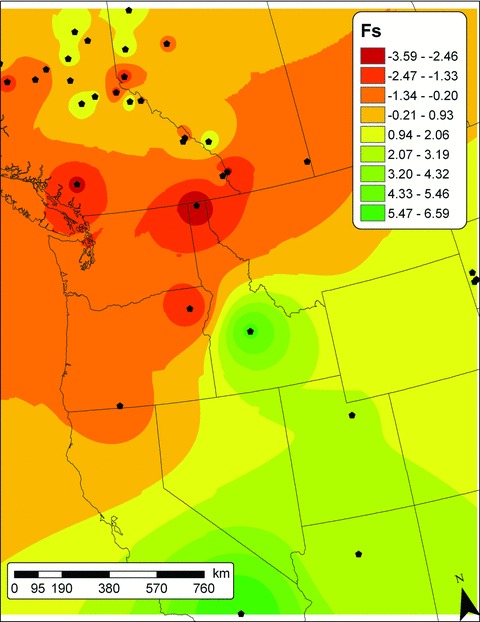
Spatially interpolated surface of range expansion (measured as Fu's [1997] *F*s) for MPB populations across western North America, generated using inverse-distance weighting in the spatial analyst extension of ArcMap9.2.

**Figure 5:**
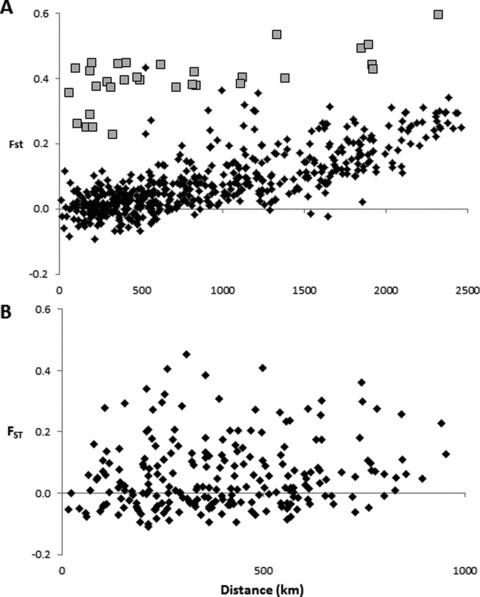
Isolation by distance plot for *D. ponderosae* across (A) western North America; and (B) our sample areas in British Columbia and Alberta. Geographic distance is significantly correlated to genetic differentiation (*F*_ST_) across the entire region (*r*= 0.64, *P*= 0.0001), though more weakly among the outbreak populations alone (*r*= 0.30, *P*= 0.0033). Comparisons with FSJ-BC are indicated by gray squares.

Among sampling areas, we found high haplotype diversity (0.756–1.000) and low nucleotide diversity (0.001–0.011) (Table 1). Nucleotide diversity declined significantly with increasing latitude (*R*^2^= 0.39, *t*_32_=–3.41 × 10^–3^, *P*= 7.47 × 10^–5^), but haplotype diversity was unrelated to either latitude or longitude (*R*^2^_latitude_= 0.05, *t*_32_= 5.94 × 10^3^, *P*= 0.19; *R*^2^_longitude_= 1.38 × 10^–3^, *t*_32_=–9.42 × 10^–5^, *P*= 0.83). There was a greater than 10-fold difference in nucleotide diversity between the southern and northern beetle populations (Fig. 2). There were minor differences between the two expansion statistics we calculated; therefore, we chose to map *F*s to illustrate patterns of demographic expansion (Fig. 4). Only two populations (BF-ID and WH) had significant signals of expansion, located in the central regions, we plotted the actual values of *F*s because they were highly correlated with the *P*-values (*r*= 0.93). The signal of expansion decreased to the north with no evidence of expansion in the south. However, when we analyzed the population groups based on their histories, we found a significant signal of expansion only for the central (*F*s =–13.40, *P*= 0.003) and no significant signal for the southern (*F*s =–7.79, *P*= 0.050) or northern groups (*F*s =–5.76, *P*= 0.107). Genetic differentiation was highly correlated with geographic distance for the combined data (*r*= 0.64, *P*= 0.0001, Fig. 5A), however this relationship was weaker when only the current outbreak samples were included (*r*= 0.30, *P*= 0.0033, Fig. 5B). There were no significant *F*_st_ comparisons among our 22 sampled populations in the outbreak region following Bonferroni correction.

## Discussion

Our assessment of mitochondrial diversity in MPB across British Columbia and Alberta indicates poorly resolved phylogenetic structure with limited genetic differentiation. Across the sampled range, we observed high haplotype diversity, but found over a 10-fold difference in nucleotide diversity between the southern and central/northern populations, resulting in a significant correlation with latitude. When combined with the data from Mock et al. (2007), we found a significant signal of isolation by distance, which agrees with their prior analysis. Based on the limited genetic structure, we hypothesize there were multiple outbreak sources in southern British Columbia in the current epidemic. Analysis with microsatellite markers also found southern British Columbia represented a single genetic population (N. G. Samarasekara et al. unpubl. ms.) with weak substructure due to the size and rapid spread of this outbreak. Although little phylogenetic structure was apparent, there were interesting patterns of diversity and expansion that can be used to make some demographic inferences. As well, these data bring to light potential limitations of phylogeographic inference for species with similar demographics.

Limited phylogenetic structure of MPB is not surprising given their population demographics. MPB development is completed most often in one year and under certain conditions in two years (Amman 1977; Safranyik et al. 2010) and following development within the host tree, adult beetles emerge and disperse (Safranyik and Carroll 2006). Dispersal is either short (among host trees, 30 m; Safranyik et al. 1992) or medium distance (between forest stands, 20 km; Furniss and Furniss 1972), although beetles have been shown to achieve long-range dispersal of >100 km in a single day (Safranyik et al. 2010). Given the dispersive nature of the MPB life cycle and the distances they travel, the potential for isolated populations and genetic signatures to develop should be limited.

Along with being dispersive, MPB also routinely experience large fluctuations in population size. Since documentation of MPB attacks began in British Columbia approximately 125 years ago, there have been four to five major outbreaks, often in different geographic areas (Taylor and Carroll 2004; Aukema et al. 2006). The level of haplotype diversity is affected by the effective population size, where a loss of diversity will result when the effective population is reduced. For outbreaking populations, there is an expectation of reduced diversity since the long-term effective population will reflect the size of the endemic phase (Motro and Thomson 1982). Estimates of population size for MPB in the endemic phase are <40 attacking females per hectare (Carroll et al. 2006), a considerably smaller population than the epidemic phase where attack densities are on average 70 beetles/m^2^ bark (Raffa and Berryman 1983). Despite this expectation, we observed very high haplotype diversity in all of our studied populations (Table 1), consistent with other species of irruptive bark beetles (Cognato et al. 1999; Anducho–Reyes et al. 2008; Ruiz et al. 2009). Based on other studies of outbreaking populations, there are two factors that likely act to maintain high diversity. First, the dispersive behavior of MPB will result in constant immigration, which can act to maintain high levels of heterozygosity (Ehrich and Jorde 2005). Second, the effective population size at the endemic phase for MPB may be high enough to maintain diversity, and/or the length of the endemic phase is not long enough for a considerable decrease in diversity, this was observed for outbreaking locust populations in Europe (Chapuis et al. 2009).

Using a phylogeographic approach, other studies have been able to assess the geographic source of outbreaking insects (Davies et al. 1999; Kobayashi et al. 2010). When multiple sources contribute to an outbreak, it will generally result in genetic population clusters (Mun et al. 2003; Kobayashi et al. 2010). Spatiotemporal analysis of outbreak data in BC indicates the current epidemic resulted from a simultaneous increase in populations in southern BC (Aukema et al. 2006), suggesting multiple sources. Yet, we did not observe evidence of population structure, rather we observed nonsignificant pair-wise *F*_ST_ comparisons and very diffuse isolation by distance among outbreak populations (Fig. 5B). In both Mun et al. (2003) and Kobayashi et al. (2010), the outbreaks were geographically separate whereas the MPB outbreak in southern BC involved multiple areas that are geographically proximate. Proximity and the dispersal capability of MPB have likely contributed to the mixing of mitochondrial lineages during the outbreak, which would be consistent with the findings of Aukema et al. (2006).

The limited degree of phylogenetic structure across a large geographic region for MPB raises an important consideration: how applicable is a phylogeographic approach to resolving the history of a highly dispersive, irruptive species? There have been a number of studies that have considered the phylogeographic structure of outbreaking insect populations (Cognato et al. 2005; Maroja et al. 2007; Mock et al. 2007; Anducho–Reyes et al. 2008; Ruiz et al. 2009) and these found limited phylogenetic structure, high haplotype diversity, low nucleotide diversity, and evidence of range expansion. For both our analysis of MPB and that of Mock et al. (2007), the signal of range expansion that we observe is most likely to be from post-Pleistocene historical processes and the more recent expansion is not detectable using traditional expansion statistics. Since record taking began in the early 1900s, MPB has been documented primarily in southern and central British Columbia (Carroll et al. 2003; Taylor and Carroll 2004), and in the past 50 years the range has increased substantially toward the north. While we were unable to detect expansion using statistical measures, we do see the relationship between haplotype diversity and latitude that is suggestive of a historical range expansion.

The magnitude of the recent outbreak, in terms of both the rapid population size increase and the geographic spread, has the potential to swamp phylogeographic signal. For example, Mock et al. (2007) sampled one site in British Columbia at Fort St. James (FSJ) in 2003 (S. Lindgren, pers. comm.). FSJ is proximate to our sample sites in Francois Lake (FL) and Mackenzie (MK), which were obtained in 2005/2006. The FSJ sample is characterized by extremely low haplotype diversity (*H*= 0.2222) relative to all of our datapoints including its nearest neighbors (*H*_FL_= 0.8909, *H*_MK_= 0.8889). This low diversity is a signature of a very recently founded population (Mayr 1942), and in 2003 this area was at the front of the MPB outbreak. Yet, within three years (equal to three generations), this signature is no longer evident in our samples. This suggests that recent population demographic change has the potential to obscure population genetic signatures. In fact, Lee et al. (2007) studied the genetic diversity of *Grosmania clavigera*, a fungal associate of MPB, and found two distinct lineages in the northern Rocky Mountains. However, more recent studies did not find any lineage diversification in a comprehensive treatment of the region (Alamouti et al. 2011; Roe et al. 2011), suggesting that rapid expansion of MPB replaced the endemic populations of fungi in the northern Rocky Mountains.

This study provides information on MPB dynamics, but we have also highlighted potential drawbacks in using phylogeographic analyses for highly dispersive species with large fluctuations in population size. This is clearly an avenue that deserves more detailed attention through model-based phylogeographic inference (Excoffier and Heckel 2006; Kuhner 2008; Beaumont et al. 2010) as the use of phylogeography in these types of applications increases (Mun et al. 2003; Cognato et al. 2005; Kim et al. 2006; Maroja et al. 2007; Mock et al. 2007; Porretta et al. 2007; Anducho–Reyes et al. 2008; Chapuis et al. 2009; Ruiz et al. 2009). The use of a single marker may affect our ability to detect accurate patterns, a common criticism of single marker studies (e.g., Zink and Barrowclough 2008; Flanders et al. 2009); however, multiple nuclear markers are not likely to contradict these results, particularly in outbreak areas that are phylogeographically unstructured. A recent population genetic analysis using microsatellites did not reveal conflicting or additional information about MPB population history (N. G. Samarasakara et al. unpubl. ms.), rather the higher resolution of microsatellite data was able to resolve subtle population structure resulting from the recent expansion (N. G. Samarasakera et al. unpubl. ms.). Also, microsatellite data may not be appropriate for phylogeographic analysis given the differences in temporal sensitivity of microsatellite and mitochondrial data (Wang 2010).

## Supporting Information - Evolutionary Analysis

**Figure S1.** Maximum-likelihood tree generated using PhyML3.0 (Guindon and Gascuel 2003) for *Dendroctonus ponderosae*across western North America with *D. jeffreyi* as the outgroup.
