# Supplementary figures and images for: Phylogeographic insights into an irruptive pest outbreak
Source: Ecol Evol. 2012 May;2(5):908–19. doi: 10.1002/ece3.102 (PMC3399157; doi:10.1002/ece3.102)

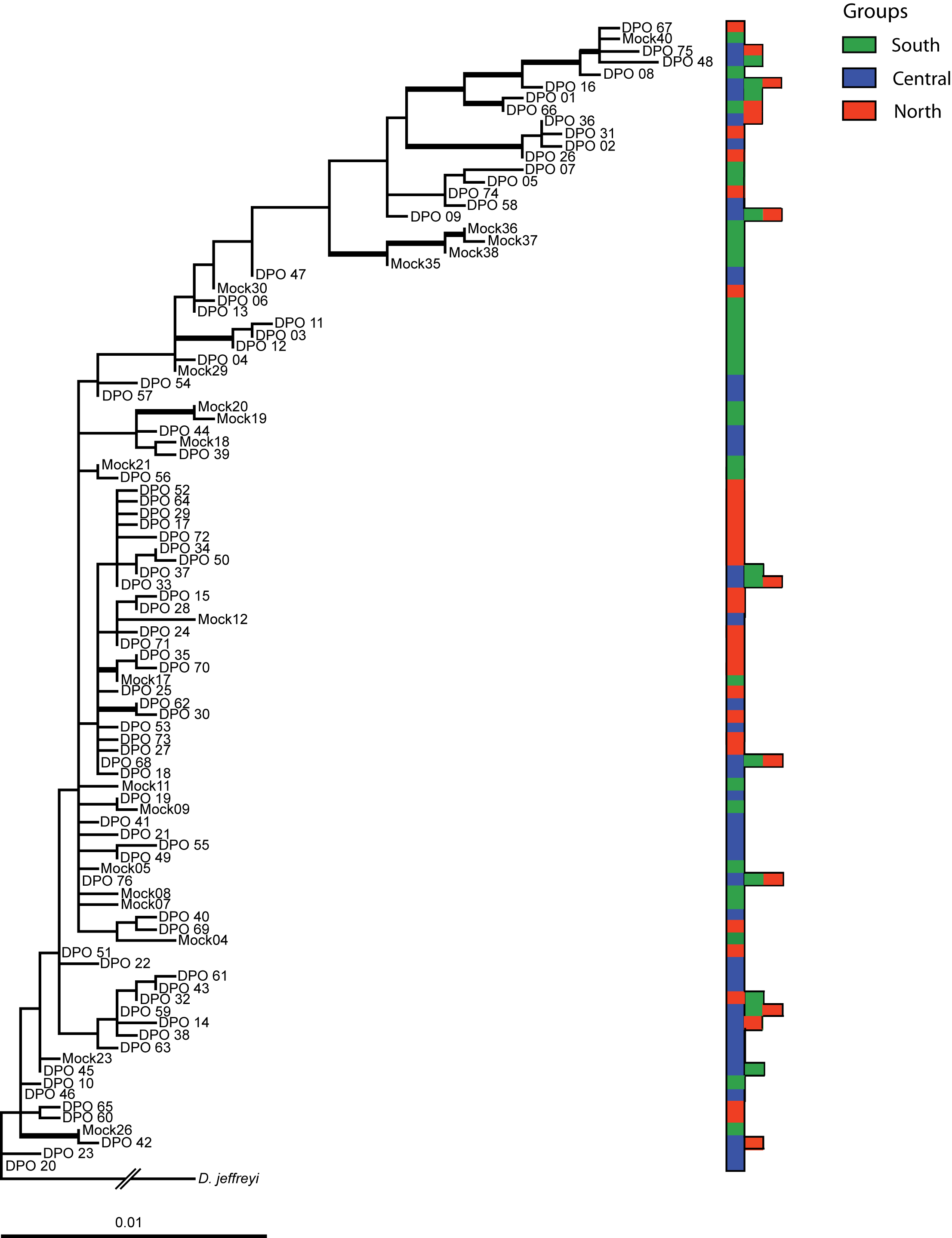

Supplement: Supplementary file 1 [file ece30002-0908-SD1.tif]
